# The optimal orthodontic displacement of clear aligner for mild, moderate and severe periodontal conditions: an in vitro study in a periodontally compromised individual using the finite element model

**DOI:** 10.1186/s12903-021-01474-7

**Published:** 2021-03-10

**Authors:** Yanning Ma, Song Li

**Affiliations:** Department of Orthodontics, School of Stomatology, Capital Medical University, No. 4, Tian Tan Xi Li, Beijing, 100050 China

**Keywords:** Clear aligner, Anterior teeth, Alveolar ridge crest, Displacement

## Abstract

**Background:**

Pathologic tooth migration (PTM) is a common complication of mild to severe periodontitis and proper orthodontic treatment is helpful to alleviate periodontal diseases. The goal of this study is to explore an optimal orthodontic displacement of clear aligner using a three-dimensional (3D) finite element model (FEM).

**Methods:**

The cone beam computed tomography (CBCT) data of a patient received invisible orthodontics without diabetes and other systemic diseases were collected. Based on the new classification scheme for periodontal diseases in 2017 (stage I: mild periodontitis, [M1]; stage II: moderate periodontitis, [M2]; stage III: severe periodontitis, [M3]), 3D-FEMs of mandible were established using MIMICS 10.0 and ABAQUS 6.5 softwares. The 3D stress distribution diagrams and stress value of the teeth (left lower incisor, left lower central incisor, right lower lateral incisor, and right lower central incisor) under three different periodontal conditions (M1, M2, and M3) with axial inclination 90° and 100° were obtained by ABAQUS 6.5.

**Results:**

The stress of anterior teeth was concentrated in the teeth neck, and became greater when the periodontal condition was worse. The stress value of anterior teeth and the strain at the top of the alveolar crest are greater as the displacement increasing. The stress value of anterior teeth and the strain at the top of the alveolar crest in axial inclination 100° are relatively great compared to those of axial inclination 90°. For patients with excessively inclined anterior teeth (such as 100°), the optimal orthodontic displacement is 0.18 mm. In order to ensure that alveolar ridge crest is not deformed, the displacement is less than 0.18 mm (strain for 0.165 mm), 0.15 mm (strain for 0.167 mm) and 0.10 mm (strain for 0.117 mm) respectively when alveolar bone is normal, resorption 1/3 or 1/3–1/2.

**Conclusions:**

The optimal orthodontic displacement for patients (M1, M2, and M3) with excessively inclined anterior teeth (axial inclination 100°) is 0.18 mm. To avoid the strain at the top of the alveolar crest, the optimal displacements for M1, M2 and M3 periodontal disease patients are less than 0.18 mm, 0.15 mm and 0.10 mm, respectively.

## Background

Pathologic tooth migration (PTM) is a common complication of mild to severe periodontitis and manifests as the inclination, elongation and fan-out of the anterior teeth [1]. The incidence of periodontal diseases among adults is as high as 76%-92% [2]. In adult patients, the speed of alveolar bone resorption under pressure is greater than that of traction and hyperplasia [3]. The impedance center of the tooth moves to the root and the torque is increased, which further aggravates the destruction of periodontal tissues [4]. Therefore, adult patients with periodontitis should be treated with light force to maintain the health and stability of the periodontal environment [5]. A 12-year follow-up experiment has shown that orthodontic treatment is no longer a contraindication for severe periodontal diseases [6]. Therefore, a comprehensive and complete orthodontic treatment should be carried out, otherwise accelerating periodontal inflammation and bone destruction.

Recent years, more attentions have been paid to the effects of clear aligner on periodontal diseases due to its aesthetics and comfort on wearers [7]. As a new type of orthodontic treatment, clear aligner can cover all the teeth and keratinized gingiva without obvious damage to the periodontal tissues [8]. Besides, the use of removable orthodontic appliance is conducive to the maintenance of oral hygiene and minimizes the risk of periodontal complication [9, 10]. There are significant differences between the use of fixed appliance and clear aligner. The fixed appliance exerts continuous light force by bonding the brackets on the tooth surface and ligating the arch wire [11], while the clear aligner applies a kind of instantaneous stress, which is about 50 to 500 times than that of fixed orthodontic loading [11, 12]. However, whether the instantaneous higher-stress has a certain impact on the periodontal tissues, or whether there is a gap in the efficiency of tooth movement due to different periodontal conditions still need to be explored. Additionally, among the various types of movement of teeth such as intrusion, extrusion, rotation, tipping, and alignment movements, intrusion movement is easier to cause tooth loosening or root absorption [13]. Therefore, it is very crucial to investigate intrusion movement in the treatment of periodontal diseases [13, 14].

The finite element model (FEM) is an engineering technique used to calculate stress and deformation developed on a geometric solid submitted to external forces [15–19]. Recent years, FEM has been widely applied in different dental fields, from fixtures to the simulation of dental movements to assess the stresses generated within the different tissue structures, such as alveolar bone, periodontal ligament, and teeth [20–23]. For instance, Crimi et al. used FEM to analyze the changes in the buccal cortical bone in patients undergoing orthodontics surgeries and indicated that there is no direct proportionality relationship between the extent of bone apposition/reabsorption and dental movement [24]. Interestingly, Cervino et al. introduced a new prosthodontic technology named Digital Smile Design, which is used in combination with FEM to improve the quality of the rehabilitations [25]. In addition, with the use of FEM, it is possible to determine loading and displacement patterns according to the appliance used [26, 27]. But relevant FEM researches on the optimal displacement of clear aligner under different periodontal-statuses are relatively rare.

In this study, we investigated the effects of anterior teeth intrusion on different periodontal conditions (stage I: mild periodontitis, [M1]; stage II: moderate periodontitis, [M2]; stage III: severe periodontitis, [M3]) using a 3D-FEM, and explored the optimal displacement of teeth when anterior teeth were intruded using clear aligner. This study is aimed to provide a direction for the use of clear aligner in clinic.

## Methods

### Establishment of mandible model

**Fig. 1:**
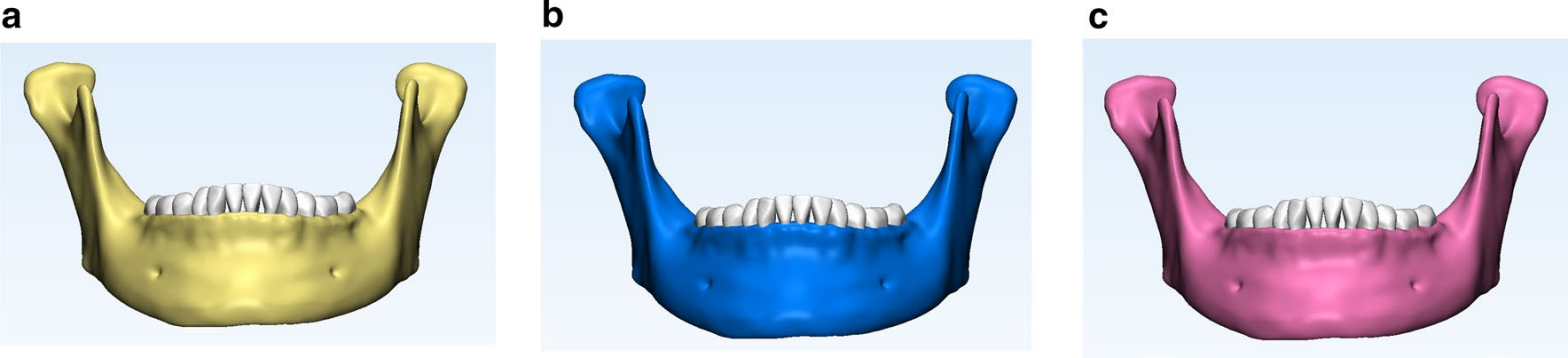
The 3D mandible models of three different periodontal conditions. **a** The 3D model of normal periodontal condition. **b** The 3D model of mild periodontal disease. **c** The 3D model of moderate periodontal disease

The Cone Beam Computed Tomography (CBCT) data (HiRes3D-Plus; Largev, Beijing, China) of a patient (female, 35 years old) with mild periodontitis received invisible orthodontics were collected. The exclusion criteria for the patient included diabetes and/or other systemic diseases that may cause progressive periodontal injury. Afterwards, based on the new classification scheme for periodontal diseases in 2017 (M1: stage I, normal alveolar bone with mild periodontitis; M2: stage II, alveolar bone resorption 1/3 with moderate periodontitis; M3: alveolar bone resorption 1/3–1/2 with severe periodontitis) [28, 29], 3D-FEMs of mandible (Fig. 1) were established using MIMICS 10.0 (Materi-alise, Leuven, Belgium) and ABAQUS 6.5 (ABAQUS. Inc, USA) softwares as previous studies described [17–19]. Though the finite element software ABAQUS 6.5, we calculated the stress of each part in the models, and obtained the 3D stress distribution diagrams and stress value of the teeth.

### Simulation of anterior teeth intrusion

To simulate of anterior teeth intrusion (left lower incisor, left lower central incisor, right lower lateral incisor, and right lower central incisor), the displacement of the teeth (0.10 mm, 0.15 mm, 0.18 mm, 0.20 mm and 0.25 mm, respectively) was preseted [14] and was used to analyze the change of stress value with two different axial inclinations of anterior teeth (90° and 100°). The stress value of normal periodontal-condition was utilized as a control.

### Structure parameters and calculation of stress

An elastic-lineal behavior and standard Young's modulus (alveolar bone: 1.40 × 10^3^ MPa; tooth: 1.96 × 10^4^ MPa; clear aligner: 528 MPa; vertical rectangular-attachment: 1.25 × 10^4^ MPa) and Poisson's ratio (alveolar bone: 0.30; tooth: 0.30; clear aligner: 0.36; vertical rectangular-attachment: 0.36) of the model were considered for all calculations as the previous studies described [30, 31]. The Von Mises comprehensive equivalent stress was selected as the main indicator to measure the stress level. Though the finite element software ABAQUS 6.5, we calculated the stress of each part in the models, and obtained the 3D stress distribution diagrams and stress value of the teeth.

## Results

### The stress distribution and stress value of the lower anterior teeth with three different periodontal conditions

**Fig. 2:**
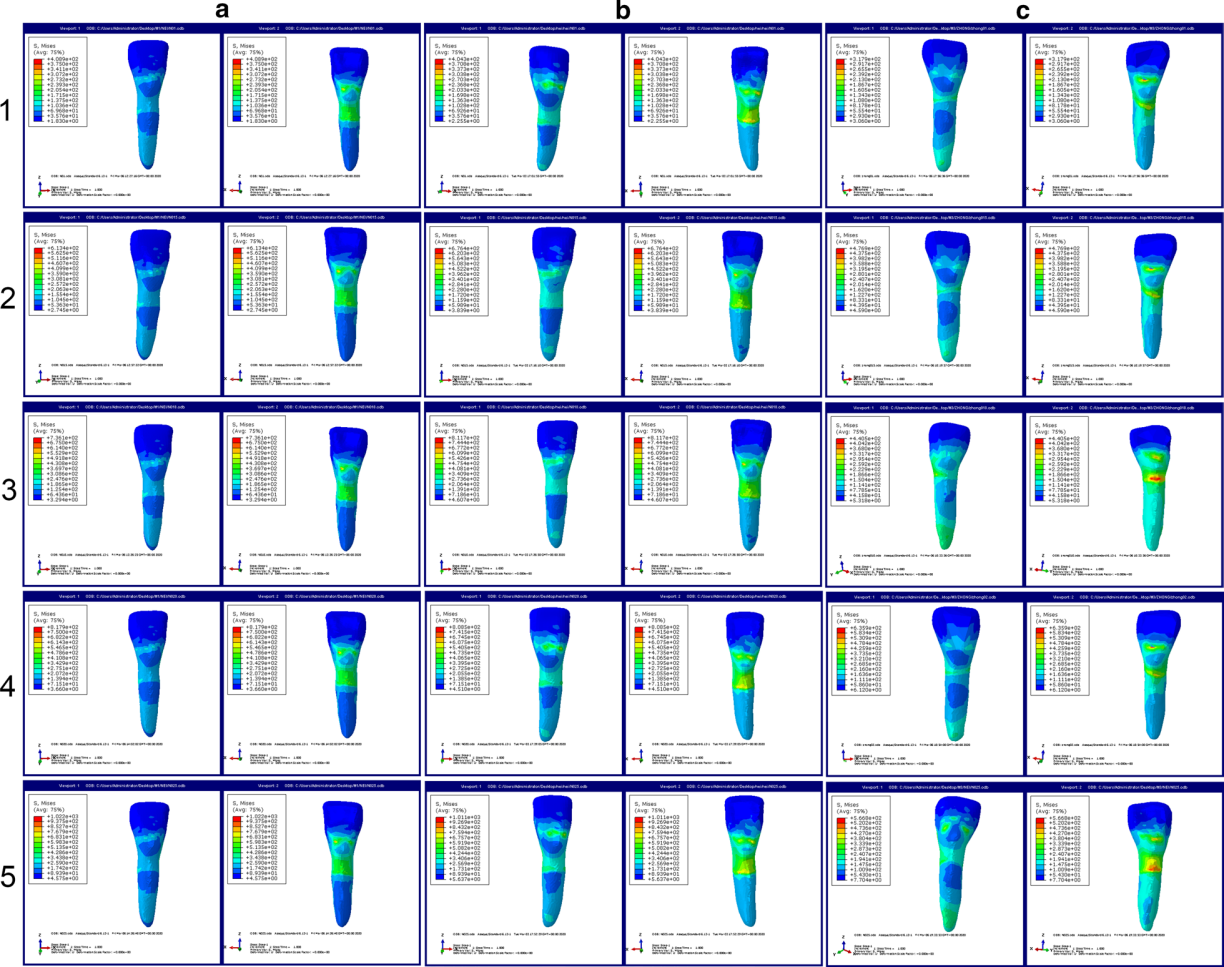
The stress distributions of the lower anterior teeth under three different periodontal conditions with axial inclination 90°. **a** The stress distributions of the lower anterior teeth under normal periodontal condition with axial inclination 90°. **b** The stress distributions of the lower anterior teeth under mild periodontal disease with axial inclination 90°. **c** The stress distributions of the lower anterior teeth under moderate periodontal disease with axial inclination 90°. 1: 0.10 mm displacement; 2: 0.15 mm displacement; 3: 0.18 mm displacement; 4: 0.20 mm displacement; 5: 0.25 mm displacement

**Fig. 3:**
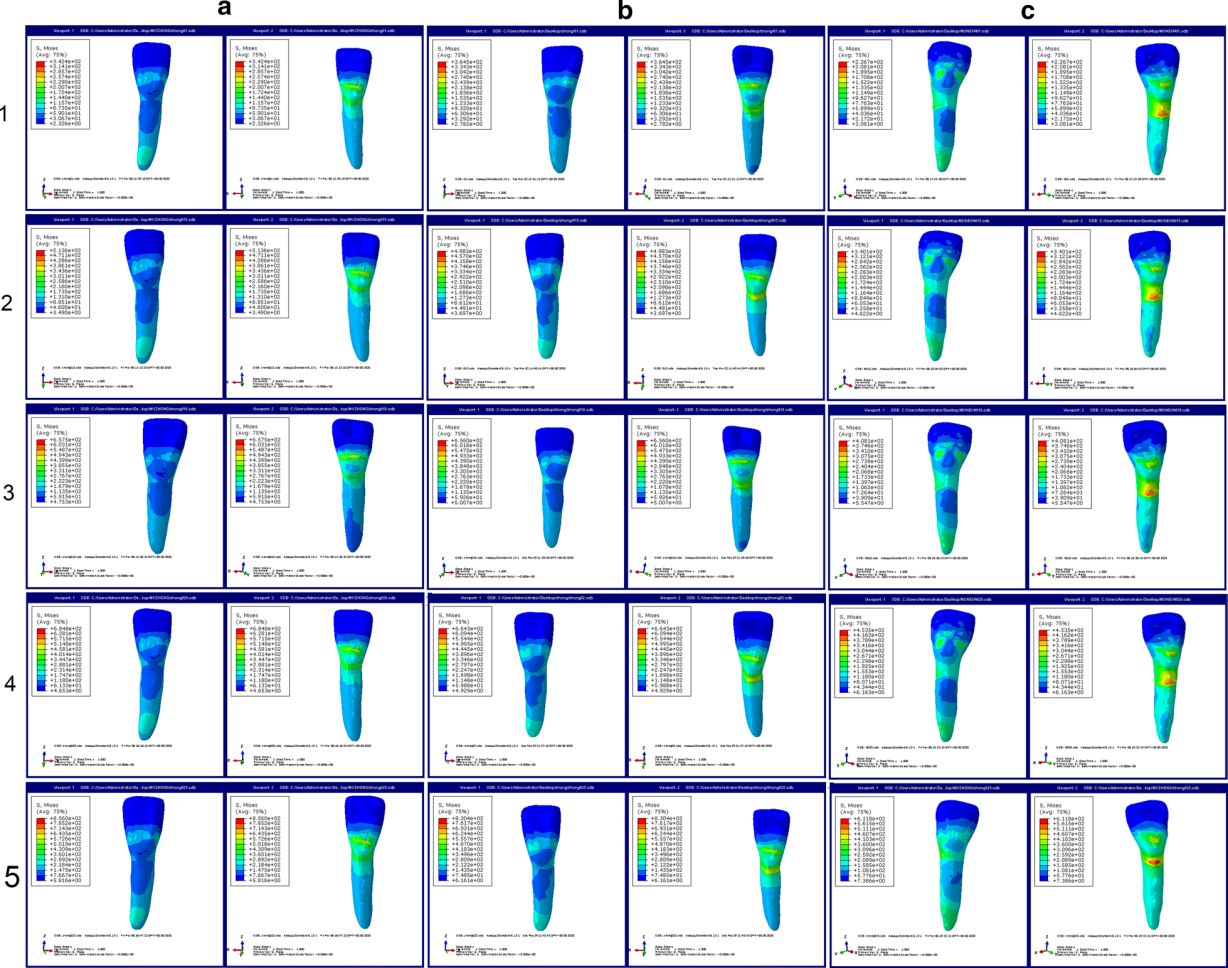
The stress distributions of the lower anterior teeth under three different periodontal conditions with axial inclination 100°. **a** The stress distributions of the lower anterior teeth under normal periodontal condition with axial inclination 100°. **b** The stress distributions of the lower anterior teeth under mild periodontal disease with axial inclination 100°. **c** The stress distributions of the lower anterior teeth under moderate periodontal disease with axial inclination 100°. 1: 0.10 mm displacement; 2: 0.15 mm displacement; 3: 0.18 mm displacement; 4: 0.20 mm displacement; 5: 0.25 mm displacement

**Fig. 4:**
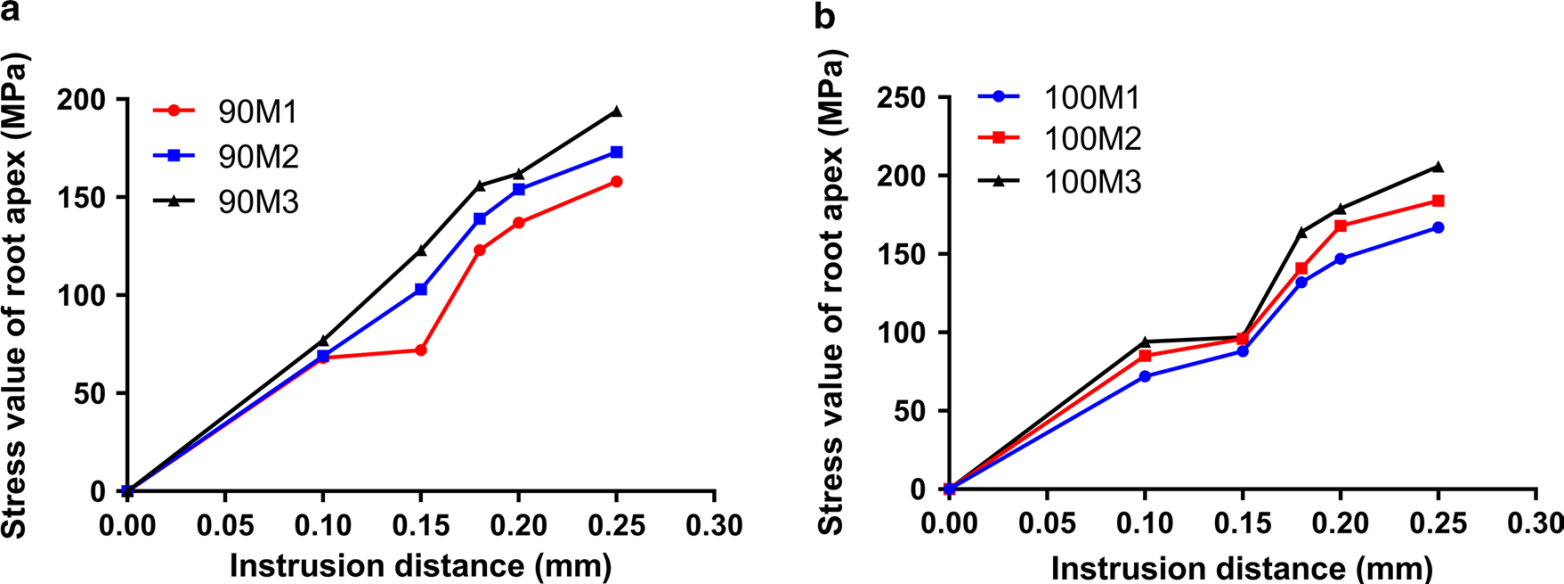
The stress values of root apex in three periodontal conditions. **a** The stress values of the lower anterior teeth under three different periodontal conditions with axial inclination 90°. **b** The stress values of the lower anterior teeth under three different periodontal conditions with axial inclination 100°

First, the stress distributions of the lower anterior teeth under three different periodontal conditions with two different axial inclinations of anterior teeth (90° and 100°) were observed (Figs. 2 and 3). We discovered that the stress of anterior teeth became stronger and was concentrated in the teeth neck when the periodontal condition was worse in the same axial inclination of anterior teeth (90° or 100°). Besides, as the displacement (0.10 mm, 0.15 mm, 0.18 mm, 0.20 mm and 0.25 mm, respectively) increasing, the stress was also more powerful. As illustrated in Fig. 4, we analyzed the stress values with quantification. The results demonstrated that the stress values (M1: 68 MPa, M2: 69 MPa, M3: 77 MPa) in 0.10 mm displacement were relatively low, whereas were increased greatly (M1: 72 MPa, M2: 103 MPa, M3: 123 MPa) starting from 0.15 mm displacement in the condition of axial inclination 90°. The stress values in 0.20 mm displacement were 137, 154, and 162 MPa (from M1 to M3), and were 158, 173, and 194 MPa (from M1 to M3) in 0.25 mm displacement (Fig. 4a). Interestingly, the stress values of anterior teeth in axial inclination 100° are relatively great compared to those of axial inclination 90°. In the condition of axial inclination 100°, the stress values of 0.10 mm were 72, 85, and 94 MPa (from M1 to M3), and next were 0.15 mm (M1: 88 MPa, M2: 96 MPa, M3: 97 MPa), 0.18 mm (M1: 132 MPa, M2: 141 MPa, M3: 164 MPa), 0.20 mm (M1: 147 MPa, M2: 168 MPa, M3: 179 MPa) and 0.25 mm (M1: 167 MPa, M2: 184 MPa, M3: 206 MPa).

### The correlation between the strain at the top of the alveolar crest and displacement

**Fig. 5:**
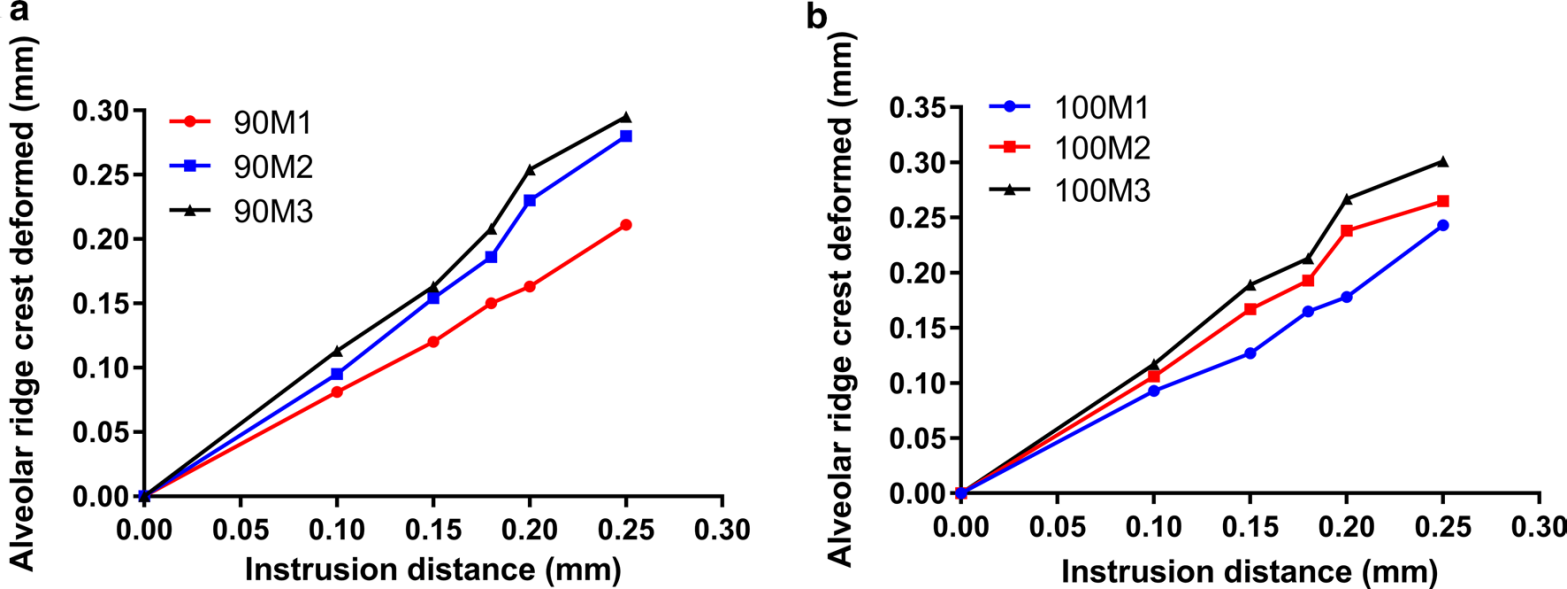
The strain at the top of the alveolar crest in three periodontal conditions. **a** the strain at the top of the alveolar crest in three periodontal conditions with axial inclination 90°. **b** The strain at the top of the alveolar crest in three periodontal conditions with axial inclination 100°

Subsequently, we analyzed the correlation between the strain at the top of the alveolar crest and displacement (0.10 mm, 0.15 mm, 0.18 mm, 0.20 mm and 0.25 mm, respectively) with two different axial inclinations of anterior teeth (90° and 100°) (Fig. 5). On the whole, we found that the alveolar ridge crest was deformed seriously as the displacement increasing in both two axial inclinations. In the terms of axial inclinations 90°, the ranges of strain at the top of the alveolar crest were 0.081–0.163 mm (0.1 mm displacement, M1: 0.081 mm, M2: 0.095 mm, M3: 0.113 mm; 0.15 mm displacement, M1: 0.120 mm, M2: 0.154 mm, M3: 0.163 mm) when the displacement was less than 0.15 mm. As the displacement increasing from 0.18 mm to 0.25 mm, the strain at the top of the alveolar crest was more visible, which ranged from 0.150 to 0.295 mm (0.18 mm displacement, M1: 0.150 mm, M2: 0.186 mm, M3: 0.208 mm; 0.2 mm displacement, M1: 0.163 mm, M2: 0.230 mm, M3: 0.254 mm; 0.25 mm displacement, M1: 0.211 mm, M2: 0.280 mm, M3: 0.295 mm) (Fig. 5a). The strain at the top of the alveolar crest was further elevated when axial inclinations changed to 100°. As shown in Fig. 5b, the ranges of the strain at the top of the alveolar crest were 0.093–0.117 (M1: 0.093 mm, M2: 0.106 mm, M3: 0.117 mm), 0.127–0.189 (M1: 0.127 mm, M2: 0.167 mm, M3: 0.189 mm), 0.165–0.213 (M1: 0.165 mm, M2: 0.193 mm, M3: 0.213 mm), 0.178–0.267 (M1: 0.178 mm, M2: 0.238 mm, M3: 0.267 mm), and 0.243–0.301 (M1: 0.243 mm, M2: 0.265 mm, M3: 0.301 mm) mm respectively when the displacement started from 0.10 to 0.25 mm.

## Discussion

The essence of clear aligner treatment is that teeth move under the force generated by the deformation of appliance [32]. This force is transmitted to the tooth root and periodontal tissues through the inner surface of the appliance, causing the tissues to remodel and move the tooth [32]. FEM is a widely used and effective method on orthodontic studies [16–19]. Geramy et al. and McGuinness et al. used FEM to analyze the stress components of maxillary central incisor and canine tooth respectively under different alveolar bone height [18, 19]. Tanne K et al. investigated the biomechanical effect of maxillary orthopedic forces on the craniofacial complex by use of FEM [17]. In the research conducted by Middleton et al. uncovered the stresses and strains within the periodontal ligament and surrounding bone, consequent to orthodontic loading of a tooth by application of a FEM [16]. Given the application of FEM in oral biomechanics and orthodontics, the optimal orthodontic displacement was explored through anterior teeth intrusion using a 3D-FEM in this study.

Stress is one of the most important factors affecting bone regeneration and absorption [14, 33, 34]. The teeth cannot move if the stress is too small, whereas too much stress may cause degeneration or even necrosis of the periodontal ligament [14, 33, 34]. Orthodontists should apply appropriate orthodontic force to achieve maximum tooth movement efficiency and minimum damage to root and periodontal tissues [13, 35]. This is especially true for invisible orthodontics, because the instantaneous force of the invisible orthodontic is much greater than that of the fixed orthodontic, and this instantaneous force may have a pathological reaction that is not conducive to tooth movement [36]. Tweed made a statistical study on Caucasians and found that under the conditions of facial coordination, stable dental arch and good masticatory function, the axial inclination is 90° [37]. The orthodontic treatment mainly depends on changing the position and inclination of the lower incisor. When the axial inclination is greater than 90° (such as 100°), the lower anterior teeth presents a status of lip inclination and the lower lip was protruding, which needs orthodontic treatment [37]. In this study, we found that as the periodontal conditions becoming worse (M1 to M3), the maximum stress values of the anterior teeth change accordingly. Meanwhile, the larger of the axial inclination, the greater of stress in anterior teeth is. We conjectured that the oversize axial inclination may increase the possibility of root damage. Besides, inadequate orthodontic treatment induces an injury of the periodontium that is clinically observed as root resorption and/or alveolar bone loss [38, 39].Therefore, it was necessary to reduce the displacement of tooth when designing tooth movement. Recent studies have demonstrated that in the process of invisible orthodontic, the displacement of each tooth is 0.20–0.40 mm [40, 41]. More importantly, the default displacement of different brands of clear aligners is usually 0.20–0.30 mm per step [40, 41]. Therefore, 0.20 mm displacement in axial inclination 90° was also considered as a control among the five displacements in this study. When the axial inclination was increased, the maximum stress value of anterior teeth should not exceed the stress value when the displacement was 0.20 mm. In axial inclination 100°, we discovered that the ranges of stress value in 0.18 mm are 132–164 MPa, which are similar to the stress value ranges of 0.20 mm in axial inclination 90° (137–162 MPa). Consequently, we inferred that for patients with excessively inclined anterior teeth (such as 100°), the optimal orthodontic displacement when using clear aligner is 0.18 mm.

We should also reasonably determine the displacement based on the strain of the alveolar bone or alveolar ridge crest. Peter et al. have analyzed the 3D-FEM of alveolar bone and revealed that in order to distribute the periodontal stress evenly, a combination of force reduction and increased moment-to-force (M/F) ratio is required [42]. A research conducted by Cobo et al. has been reported that the height of the alveolar bone can directly or indirectly affect the periodontal stress distribution, and the magnitude of the change is positively related to the degree of influence [43]. In this study, the strain at the top of the alveolar crest with different displacement (0.10 mm, 0.15 mm, 0.18 mm, 0.20 mm and 0.25 mm, respectively) under two different axial inclinations of anterior teeth (90° and 100°) was explored. Likewise, 0.20 mm in 90M1 was used as the control group and the strain at the top of the alveolar crest was 0.163 mm. We found that the displacement must be not exceeding 0.15 mm when alveolar bone resorption is 1/3 or 1/3–1/2. In axial inclination 100°, we discovered that in order to ensure that alveolar ridge crest is not deformed, the displacement is less than 0.18 mm (strain for 0.165 mm), 0.15 mm (strain for 0.167 mm) and 0.10 mm (strain for 0.117 mm) respectively when alveolar bone is normal, resorption 1/3 or 1/3–1/2. All the results implied that the stress on the teeth and the strain at the top of the alveolar crest should be taken into account at the same time when presetting displacement in orthodontic treatment.

However, there are some limitations in this study. First, this study is focused on four of the anterior teeth, and posterior tooth movement remains unclear. Second, tooth movements are influenced by the patient’s age, periodontal support, root length, and bone density, and large sample size is necessary. Third, tooth movement is also affected by periodontal tissue remodeling, action time, strength attenuation, and oral and maxillofacial muscle occlusion. The results of preliminary finite element need to be used in clinical practice to verify its efficacy.

## Conclusions

In summary, we explored the optimal orthodontic displacement to minimize the adverse effects of the clear aligner on periodontal tissues under laboratory conditions. However, the structure of oral cavity is extremely complex and there may be other factors to affect the treatment effect of clear aligner. Even so, we also hope our findings will improve orthodontic treatment with clear aligners.

## Data Availability

The datasets used and/or analysed during the current study are available from the corresponding author on reasonable request.

## Abbreviations

CBCT: cone beam computed tomography
PTM: pathologic tooth migration
